# Supplementary material for: Proteome-wide and matrisome-specific alterations during human pancreas development and maturation
Source: Nat Commun. 2021 Feb 15;12:1020. doi: 10.1038/s41467-021-21261-w (PMC7884717; doi:10.1038/s41467-021-21261-w)
Supplement: Supplementary file 1 — Supplementary Information [file 41467_2021_21261_MOESM1_ESM.pdf]

Supplementary Information

Proteome-wide and matrisome-specific alterations during human pancreas development and maturation

Zihui Li[1,#], Daniel M. Tremmel[2,#], Fengfei Ma[3], Qinying Yu[3], Min Ma[3], Daniel G. Delafield[1], Yatao Shi[3], Bin Wang[3], Samantha A. Mitchell[2], Austin K. Feeney[2], Vansh S. Jain[2], Sara Dutton Sackett[2], Jon S. Odorico[2*], Lingjun Li[1,3*]

[1]Department of Chemistry, University of Wisconsin-Madison, Madison, WI, USA.

[2]Division of Transplantation, Department of Surgery, School of Medicine and Public Health, University of Wisconsin-Madison, Madison, WI, USA.

[3]School of Pharmacy, University of Wisconsin-Madison, Madison, WI, USA.

[#]These authors contributed equally: Zihui Li, Daniel M. Tremmel.

[*]Corresponding authors: jon@surgery.wisc.edu, lingjun.li@wisc.edu

Supplementary Figures

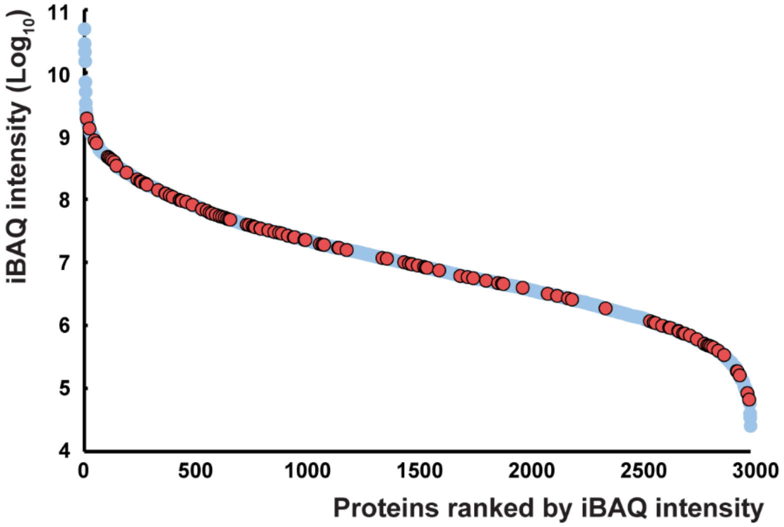

Supplementary Fig. 1 Dynamic intensity range of identified proteins in adult groups. Proteins identified in young and older adult groups are ranked and plotted from high to low based on iBAQ (Intensity Based Absolute Quantification) intensities. All quantified ECM proteins are highlighted in closed red circles. Source data are provided as a Source Data file.

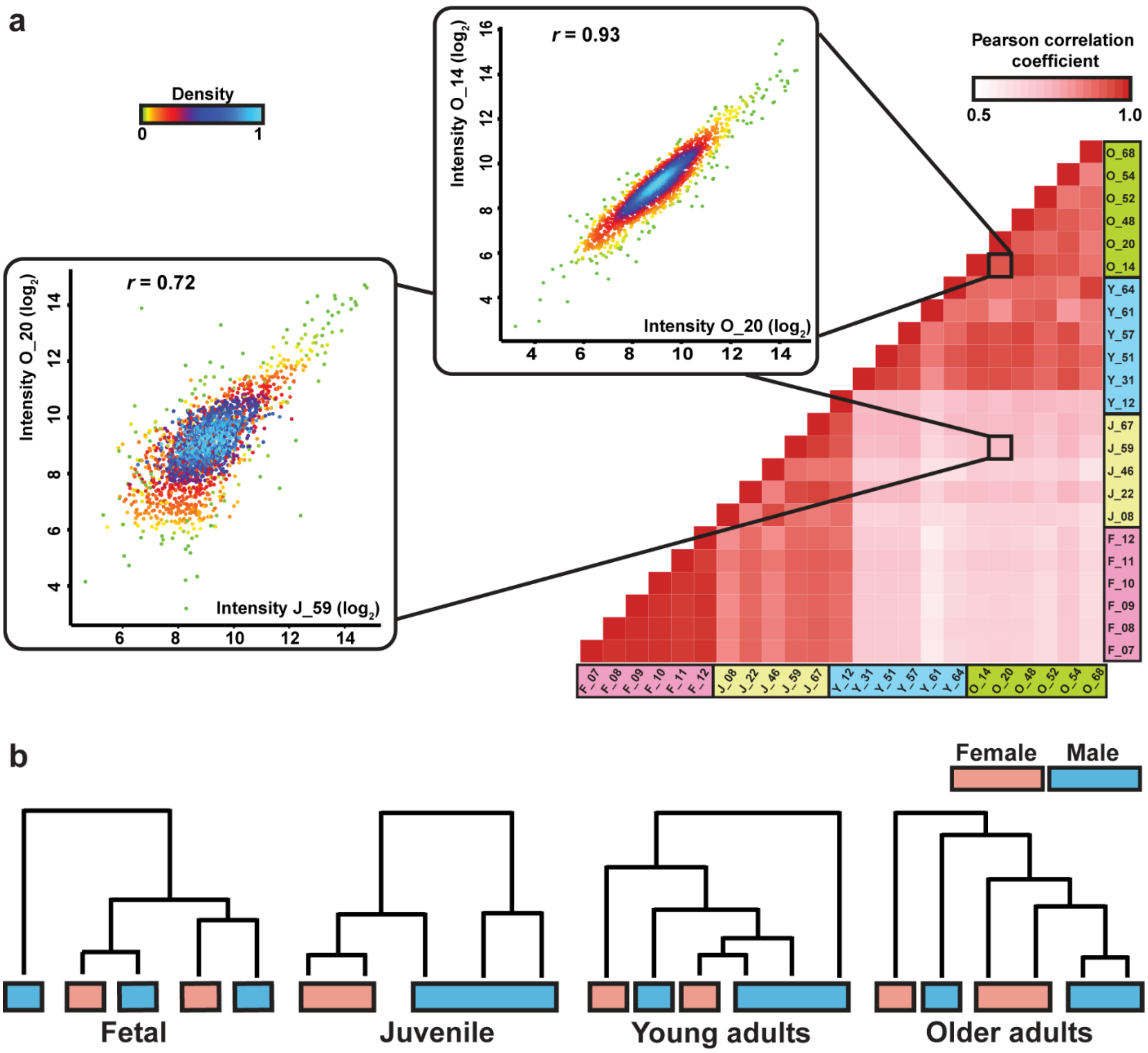

Supplementary Fig. 2 Correlation between samples and gender differences across developmental groups. a) Pearson correlation analysis of protein intensity in all samples from four age groups. Color coding of each box in the heatmap indicates the Pearson correlation coefficient between the column- and row-indexed samples. Density plots illustrate the intensity correlation of two representative pairs with a good correlated one from two samples in the same age group and a poor correlated one from two samples at different stages. **b)** Dendrograms generated from hierarchical clustering in different groups.

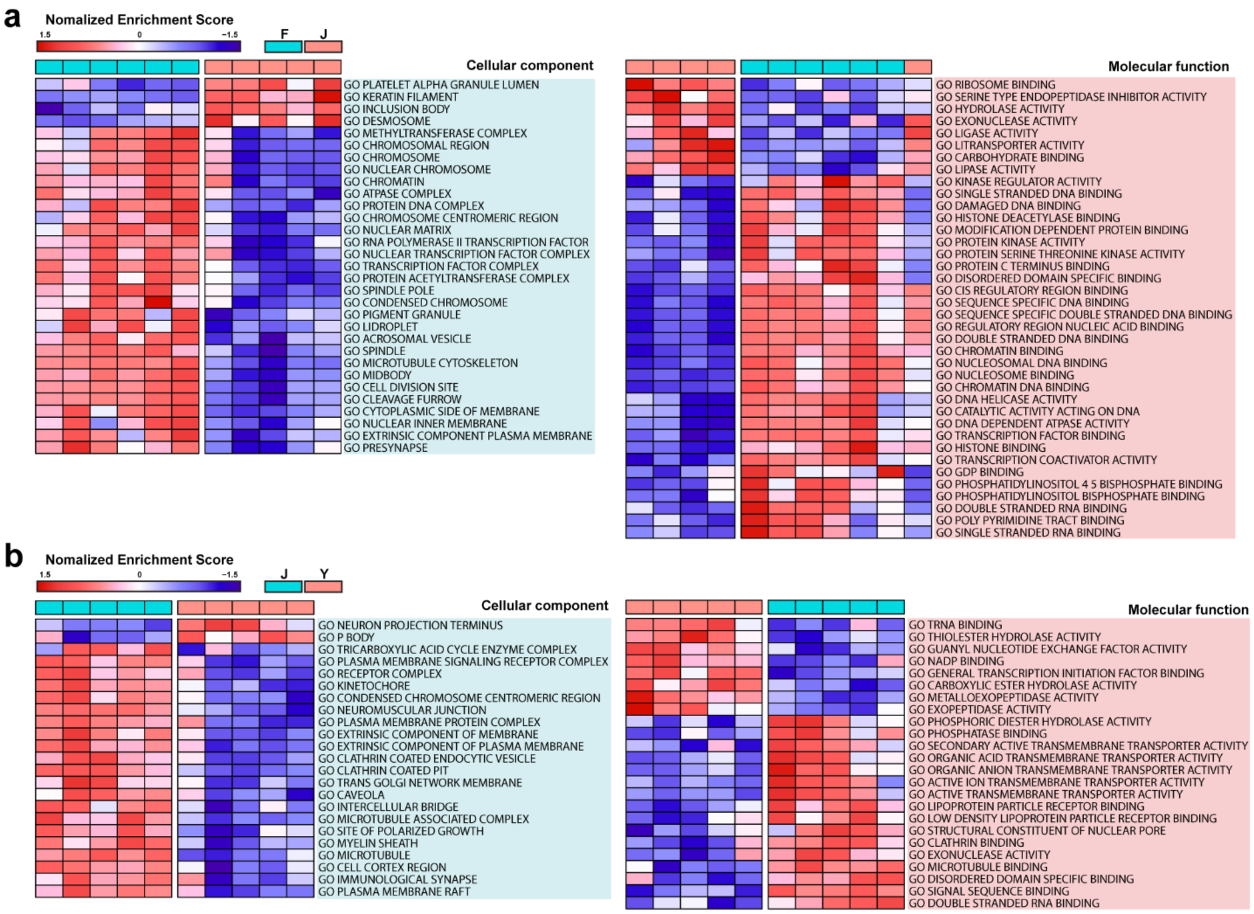

Supplementary Fig. 3 GSVA analysis in juvenile compared to fetal and young adult compared to juvenile. GSVA analysis showing the significantly changed (two-sided *t* test, p value < 0.05, p values were adjusted by Benjamini-Hochberg correction for multiple comparisons) cellular components and molecular functions in juvenile versus fetal (**a**) and young adult versus juvenile (**b**). Color coding of the heatmaps indicates normalized enrichment score in each sample. A full list of enriched terms including biological processes and transcription factor targets is available in **Supplementary Data 6**.

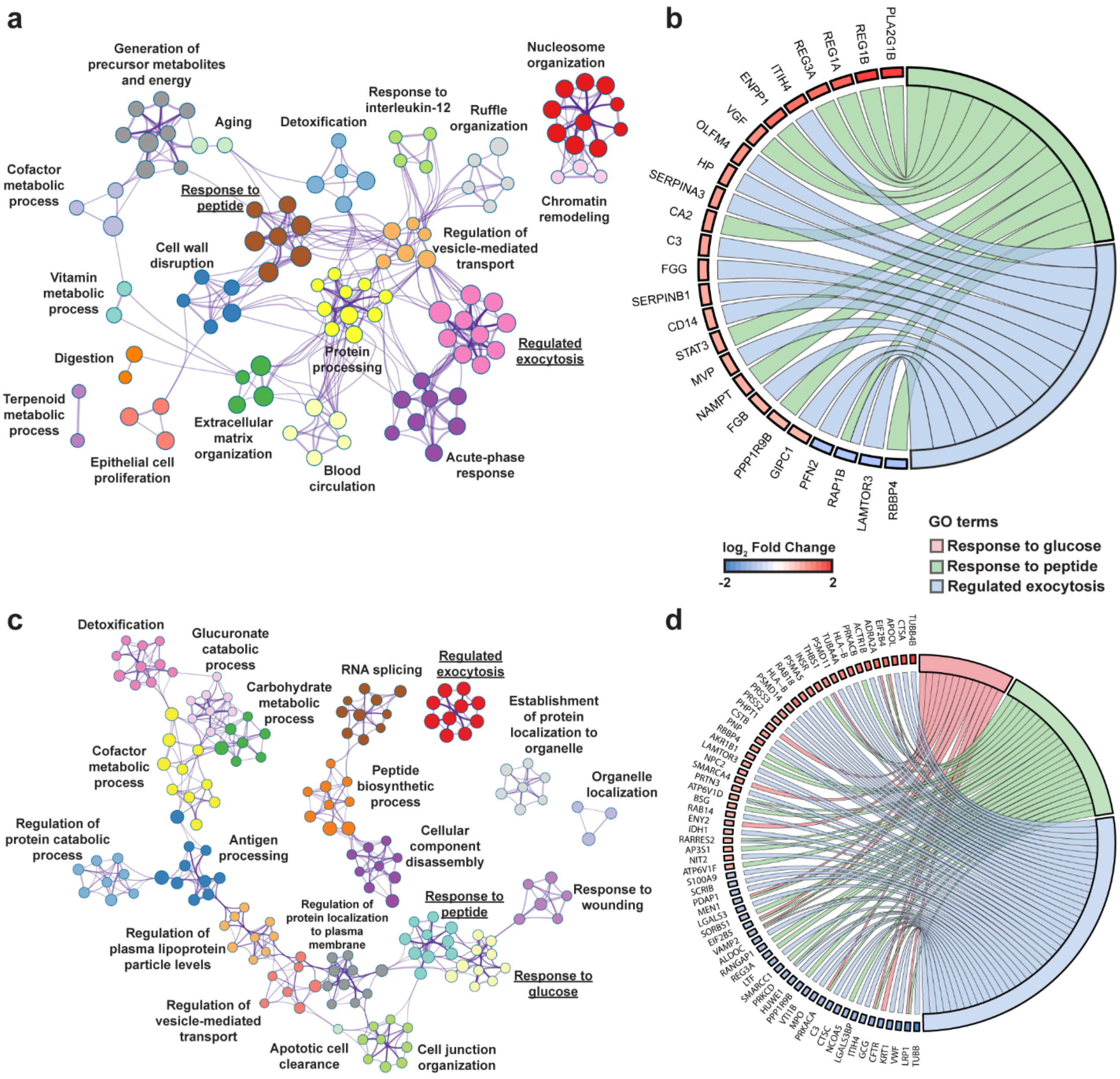

Supplementary Fig. 4 Profiling differences in juvenile compared to fetal and young adult compared to juvenile. a) Network of biological processes enriched from significantly changed proteins (two-sided t test, p value < 0.05, p values were adjusted by Benjamini-Hochberg correction for multiple comparisons) in juvenile versus fetal. Each node refers to an enriched term and different terms are grouped into clusters based on their similarities while the most statistically significant term represents the cluster name. **b)** Proteins to biological process linkages in juvenile versus fetal. Genes are linked via ribbons to their assigned terms and blue-to-red coding next to the selected genes indicates their fold changes. **c)** Network of biological processes enriched from significantly changed proteins (two-sided t test, p value < 0.05, p values were adjusted by Benjamini-Hochberg correction for multiple comparisons) in young adult versus juvenile. **d)** Proteins to biological process linkages in young adult versus juvenile.

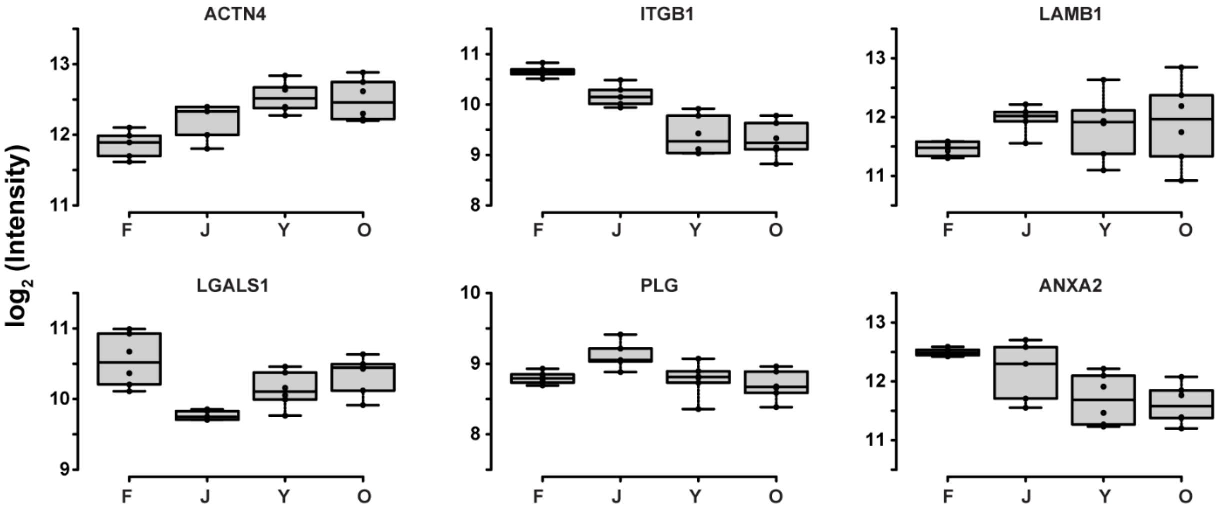

Supplementary Fig. 5 Protein expression level changes of previously reported pancreatic cancer biomarkers. Box plots showing expression levels of selected proteins at different developmental stages. Dots within boxes indicate replicate data points (F,Y,O: N=6 donors per group; J: N=5 donors). All boxplots indicate median (center line), 25th and 75th percentiles (b ounds of box), and minimum and maximum (whiskers). F, fetal; J, juvenile; Y, young adult; O, older adult. Source data are provided as a Source Data file.

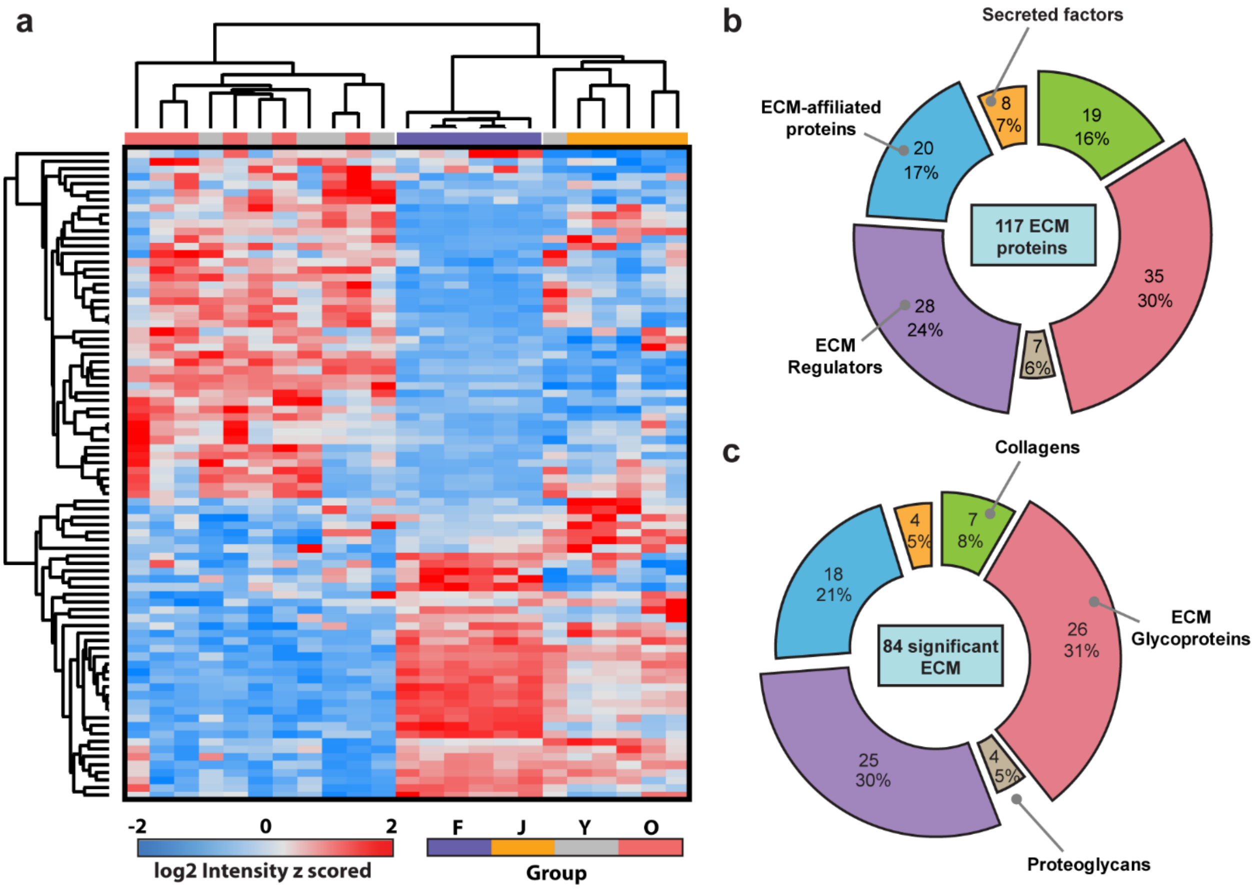

Supplementary Fig. 6 ECM profile alterations across various developmental stages. a) Hierarchical clustering of DiLeu reporter ion intensities of 84 significantly changed ECM proteins (one-way ANOVA, FDR 0.05). **b)** Pie chart showing the number and proportion of each category of all quantified ECM proteins. **c)** Pie chart showing the number and proportion of each category of significantly changed ECM proteins in **a**.

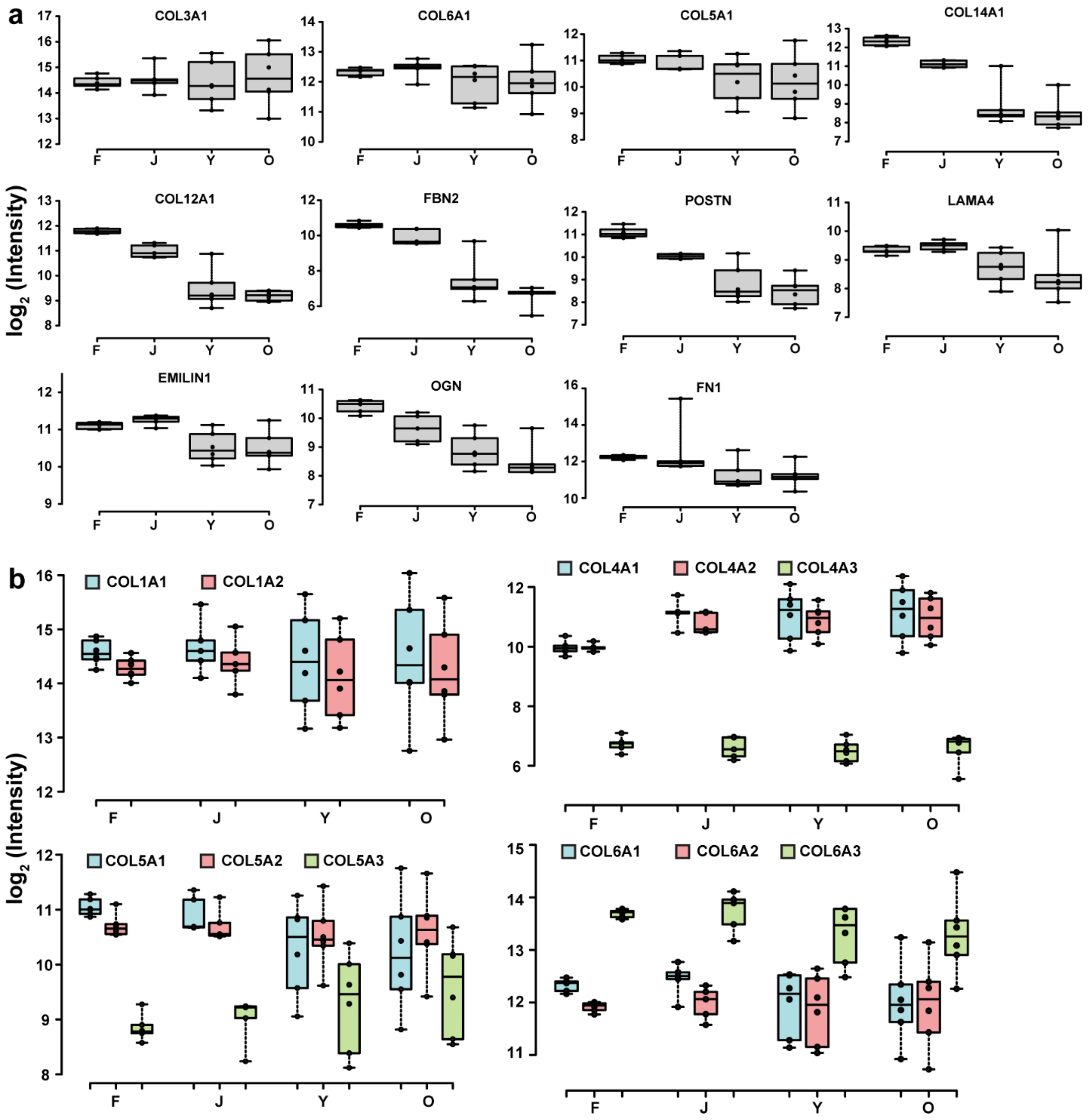

Supplementary Fig. 7 ECM remodeling of human pancreata across developmental stages. Box plots showing expression levels of selected ECM proteins (**a**) and different chains of the same collagen molecule (**b**) at different developmental stages. Dots within boxes indicate replicate data points (F,Y,O: N=6 donors per group; J: N=5 donors). All boxplots indicate median (center line), 25th and 75th percentiles (bounds of box), and minimum and maximum (whiskers). F, fetal; J, juvenile; Y, young adult; O, older adult. Source data are provided as a Source Data file.

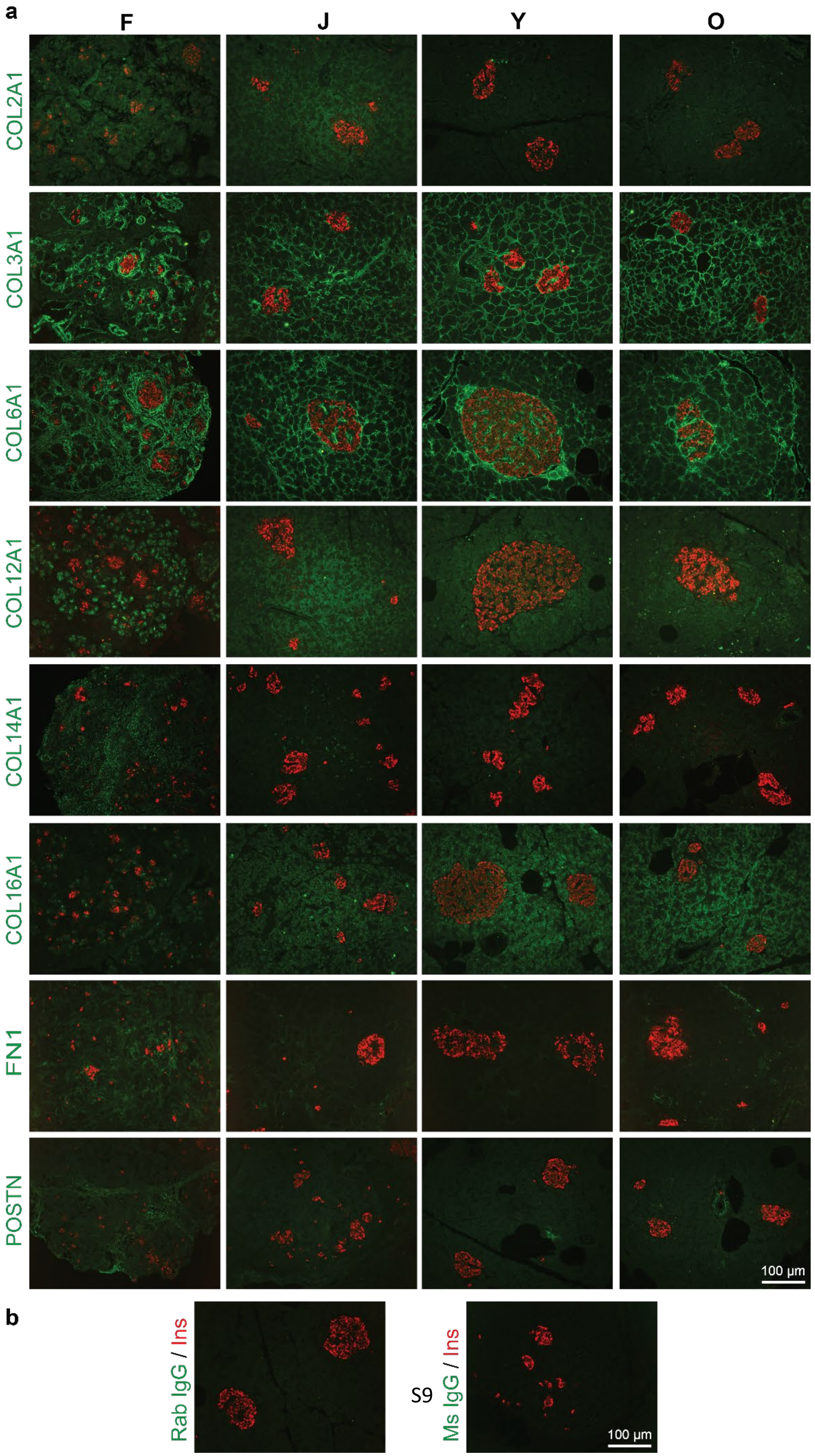
a
F
J
Y
O
COL2A1
COL3A1
COL6A1
COL12A1
COL14A1
COL16A1
FN1
POSTN
100 µm
b
Rab IgG / Ins
Ms IgG / Ins
100 µm

S9

Supplementary Fig. 8 Visualizing ECM proteins in human pancreata across developmental stages. a) Immunofluorescent images of ECM proteins (green) co-stained with insulin (red) in fetal (F), juvenile (J), young adult (Y) and older adult (O) pancreata. Qualitative trends in protein levels corroborate MS data. Representative images are shown, images were taken for N=3 donors per developmental group. **b)** IgG isotype control images with rabbit or mouse IgG (green) indicate low levels of non-specific signal, N=3 donors. Scale bar = 100 microns.

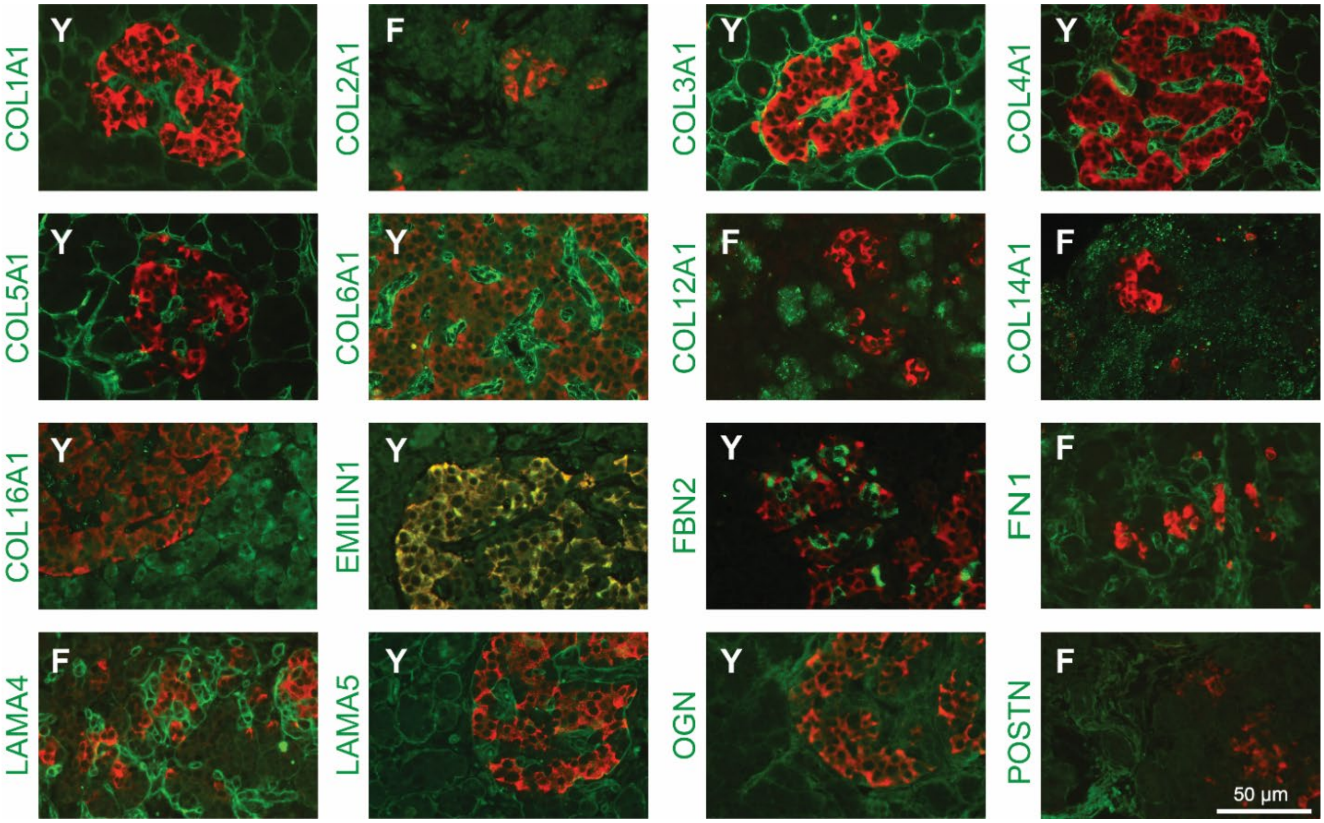

Supplementary Fig. 9 Cellular localization of ECM proteins in pancreatic islets. Enlarged images of immunofluorescent staining for ECM proteins (green) within human pancreatic islets (insulin = red). Images represent either fetal (F) or young adult (Y) donors as indicated, selected based on which age had higher intensity staining for each protein. Images clearly show differences in subcellular localization; most ECM proteins are expressed extracellularly while some proteins (such as EMILIN1 and FBN2) appear to be expressed intracellularly. Scale bar = 50 microns. Representative images shown, from N=3 donors per developmental group.

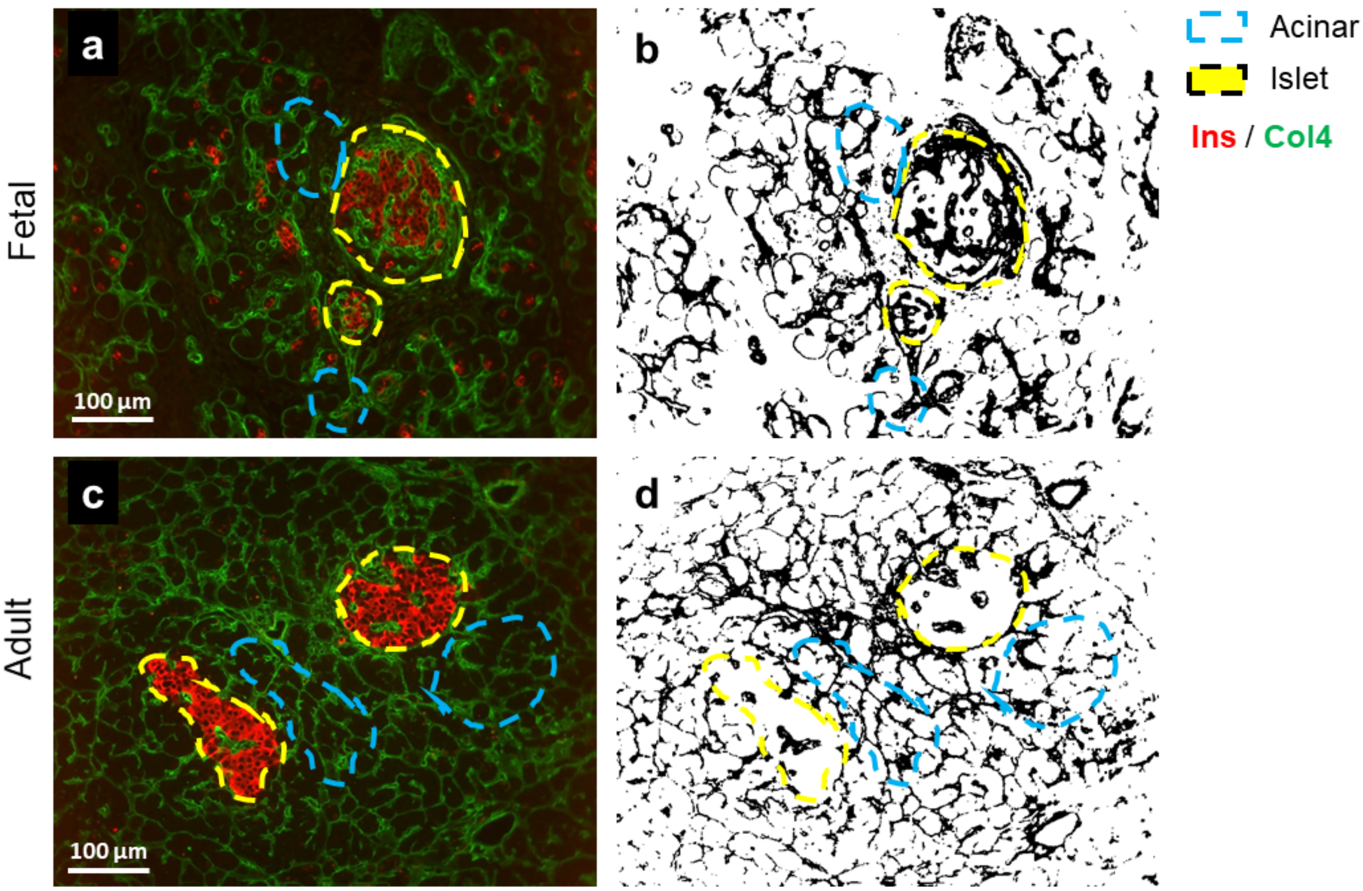

Supplementary Fig. 10 Method for quantification of Islet/Acinar Ratios. Immunofluorescent images of ECM proteins (green) co-stained with insulin (red) were analyzed using ImageJ software by tracing islets (red stain, yellow dashed line) and acinar (not red, morphologically determined, blue dashed line) in the original images **(a**, **c)**, and measuring the intensity of the ECM signal on adjusted binary images **(b**, **d)**. Representative images of fetal and adult tissue are shown for comparison. Scale = 100 microns. Representative images shown, from N=3 donors per developmental group.

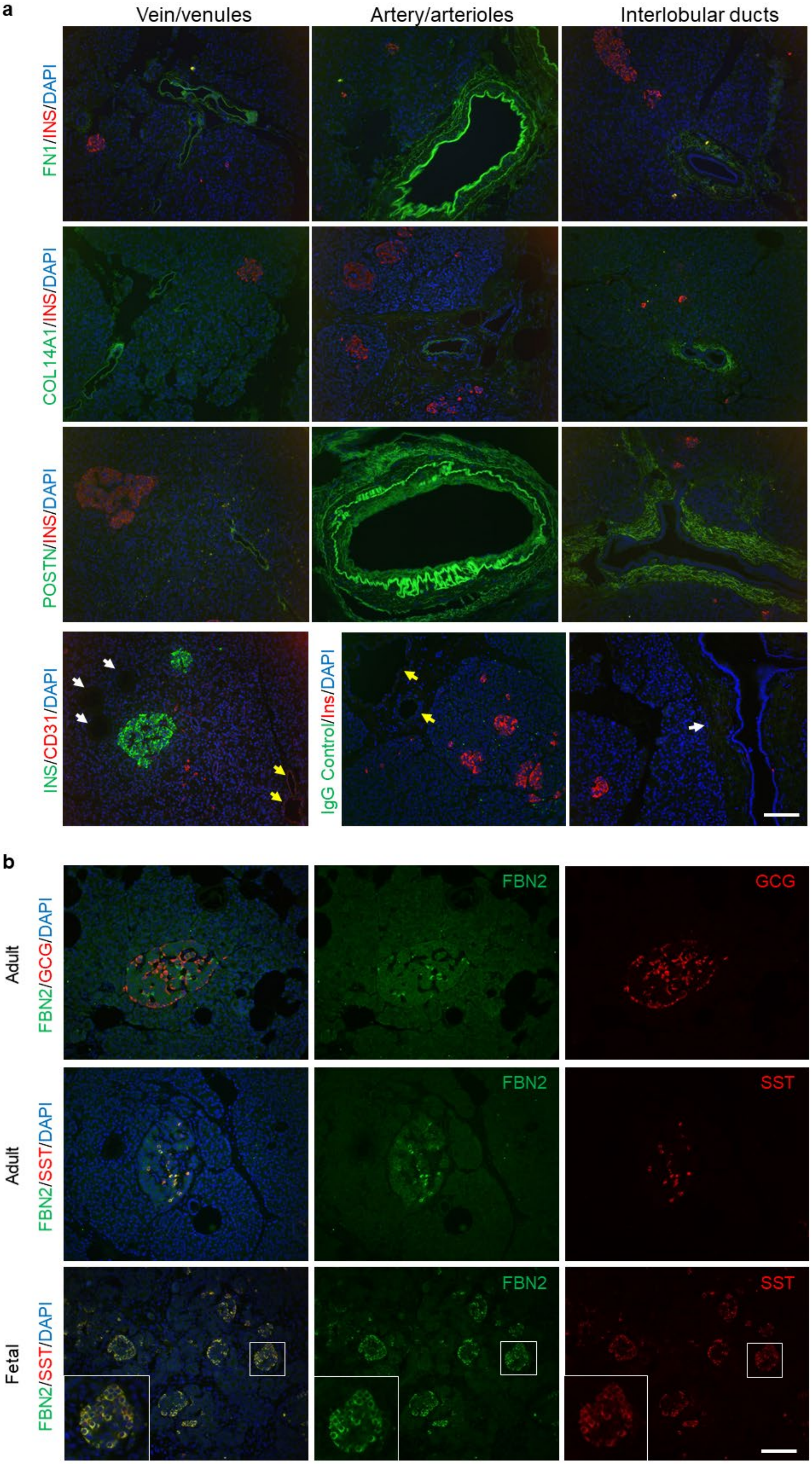
a
Vein/venules
Artery/arterioles
Interlobular ducts
FN1/INS/DAPI
COL14A1/INS/DAPI
POSTN/INS/DAPI
INS/CD31/DAPI
IgG Control/Ins/DAPI
b
FBN2
GCG
SST
Adult
Fetal
FBN2/GCG/DAPI
FBN2/SST/DAPI

Supplementary Fig. 11 Localization of ECM proteins in specific regions of the pancreas. a) Immunofluorescent staining for ECM proteins (FN1, COL14A1, POSTN = green) which had very low levels of signal in islets (Insulin = red) and acinar regions, but relatively high total protein content. These proteins were mainly found to be expressed in vessels and ducts. Control images indicate low levels of autofluorescence in the ducts (white arrows) and vessels (identified in red with positive CD31 staining; yellow arrows) when stained with an insulin antibody in green, or when stained with an IgG isotype control. **b)** Immunofluorescent staining of endocrine markers (GCG, alpha cells = red) (SST, delta cells = red) indicating the at FBN2 co-localizes with delta cells, in both fetal and adult islets. Scale bars = 100 microns. Representative images shown, from N=3 donors per developmental group.
