# Supplementary material for: Proteome-wide and matrisome-specific alterations during human pancreas development and maturation
Source: Nat Commun. 2021 Feb 15;12:1020. doi: 10.1038/s41467-021-21261-w (PMC7884717; doi:10.1038/s41467-021-21261-w)
Supplement: Supplementary file 3 — Description of Additional Supplementary Files [file 41467_2021_21261_MOESM3_ESM.docx]

**File Name:** Supplementary Data 1

**Description:** Donor information.

**File Name:** Supplementary Data 2

**Description:** List of all identified proteins.

**File Name:** Supplementary Data 3

**Description:** List of all quantified proteins. Log_2_ DiLeu reporter ion intensities of each protein in all samples are shown. Significantly changed proteins among age groups (one-way ANOVA, FDR 0.05) are highlighted in orange. Protein accession, *p* values and log_2_(fold change) for all pairwise comparisons are shown. Significantly down-regulated proteins (two-sided *t* test, *p* < 0.05, FC < 0.5) are highlighted in green and up-regulated ones (two-sided *t* test, *p* < 0.05, FC > 2) in red.

**File Name:** Supplementary Data 4

**Description:** List of all identified ECM proteins. Proteins common to all groups (quantified) are highlighted in yellow.

**File Name:** Supplementary Data 5

**Description:** List of all quantified ECM proteins. Log_2_ DiLeu reporter ion intensities of each protein in all samples are shown. Significantly changed proteins among age groups (one-way ANOVA, FDR 0.05) are highlighted in orange. Protein accession, *p* values and log_2_(fold change) for all pairwise comparisons are shown. Significantly down-regulated proteins (two-sided *t* test, *p* < 0.05, FC < 0.5) are highlighted in green and up-regulated ones (two-sided *t* test, *p* < 0.05, FC > 2) in red.

**File Name:** Supplementary Data 6

**Description:** List of significantly changed (two-sided *t* test, *p* < 0.05) cellular components, molecular functions, biological processes and transcription factor targets in J *vs*. F and Y *vs.* J in GSVA analysis.

**File Name:** Supplementary Data 7

**Description:** List of core ECM proteins sorted by abundance in each developmental group. Matrisome categories are highlighted in different colors. Significantly changed proteins among age groups (one-way ANOVA, FDR 0.05) are highlighted in orange.

**File Name:** Supplementary Data 8

**Description:** Antibody information used for IF staining.
