# Supplementary material for: Proteome-wide and matrisome-specific alterations during human pancreas development and maturation
Source: Nat Commun. 2021 Feb 15;12:1020. doi: 10.1038/s41467-021-21261-w (PMC7884717; doi:10.1038/s41467-021-21261-w)
Supplement: Supplementary file 12 — Reporting Summary [file 41467_2021_21261_MOESM12_ESM.pdf]

nature research

Corresponding author(s): Lingjun Li and Jon Odorico

Last updated by author(s): Jan 14, 2021

Reporting Summary

Nature Research wishes to improve the reproducibility of the work that we publish. This form provides structure for consistency and transparency in reporting. For further information on Nature Research policies, see our Editorial Policies and the Editorial Policy Checklist.

Statistics

For all statistical analyses, confirm that the following items are present in the figure legend, table legend, main text, or Methods section.

n/a	Confirmed	
☐	☒	The exact sample size (*n*) for each experimental group/condition, given as a discrete number and unit of measurement
☐	☒	A statement on whether measurements were taken from distinct samples or whether the same sample was measured repeatedly
☐	☒	The statistical test(s) used AND whether they are one- or two-sided<br>*Only common tests should be described solely by name; describe more complex techniques in the Methods section.*
☐	☒	A description of all covariates tested
☐	☒	A description of any assumptions or corrections, such as tests of normality and adjustment for multiple comparisons
☐	☒	A full description of the statistical parameters including central tendency (e.g. means) or other basic estimates (e.g. regression coefficient) AND variation (e.g. standard deviation) or associated estimates of uncertainty (e.g. confidence intervals)
☐	☒	For null hypothesis testing, the test statistic (e.g. *F*, *t*, *r*) with confidence intervals, effect sizes, degrees of freedom and *P* value noted<br>*Give P values as exact values whenever suitable.*
☒	☐	For Bayesian analysis, information on the choice of priors and Markov chain Monte Carlo settings
☒	☐	For hierarchical and complex designs, identification of the appropriate level for tests and full reporting of outcomes
☐	☒	Estimates of effect sizes (e.g. Cohen's *d*, Pearson's *r*), indicating how they were calculated

Our web collection on statistics for biologists contains articles on many of the points above.

Software and code

Policy information about availability of computer code

Data collection	For LC-MS/MS acquisition: Thermo Scientific Xcalibur 4.2 with Foundation 3.1 SP5, Orbitrap Fusion Lumos Tune Application 3.1, Thermo Scientific SII for Xcalibur 1.4<br>For IF staining: Images were collected using AxioVision version 4.8.2.0 software, and analyzed with standard packages in ImageJ software
Data analysis	Proteome Discoverer (v2.1), Perseus (v1.6.0.7), MaxQuant (v1.5.2.8), Metascape (v3.5), Cytoscape (v3.7.1), R (v3.6.1), R package ggplot, R package GOplot, R package Limma, Microsoft Excel 2016 under Windows 10 Microsoft Co., ImageJ (1.53c), Prism 6 (v6.07)

For manuscripts utilizing custom algorithms or software that are central to the research but not yet described in published literature, software must be made available to editors and reviewers. We strongly encourage code deposition in a community repository (e.g. GitHub). See the Nature Research guidelines for submitting code & software for further information.

Data

Policy information about availability of data

All manuscripts must include a data availability statement. This statement should provide the following information, where applicable:

- Accession codes, unique identifiers, or web links for publicly available datasets
- A list of figures that have associated raw data
- A description of any restrictions on data availability

The mass spectrometry proteomics data have been deposited to the ProteomeXchange Consortium via the PRIDE partner repository with the dataset identifier PXD020130 (https://www.ebi.ac.uk/pride/archive/projects/PXD020130). Source data are provided with this paper. An online searchable web application "Matrisome and proteome database of human pancreas" is also available at https://nc-webapp.herokuapp.com/, which enables custom searching of all quantified proteins and supports download of all proteomics datasets in this study. Full IF staining results are available from the authors upon reasonable requests. H. sapiens database used for proteomics data searching was downloaded from Uniprot (https://www.uniprot.org/). Human matrisome dataset used for ECM protein matching

was downloaded from “the Matrisome Project” (http://matrisomeproject.mit.edu/).

Field-specific reporting

Please select the one below that is the best fit for your research. If you are not sure, read the appropriate sections before making your selection.

- [x] Life sciences
- [ ] Behavioural & social sciences
- [ ] Ecological, evolutionary & environmental sciences

For a reference copy of the document with all sections, see nature.com/documents/nr-reporting-summary-flat.pdf

Life sciences study design

All studies must disclose on these points even when the disclosure is negative.

Sample size	No sample-size calculation was performed. Sample sizes were decided based on previous publications (PMID: 19053145; PMID: 28675934; PMID: 31484774; PMID: 20551380; PMID: 24436468; PMID: 28319050), experience and common standards in similar field for calculating statistical significance and also dependent on donor availability. Multiple tests and analyses were performed as described in the manuscript to ensure the samples are representative and results are conclusive.
Data exclusions	No data was excluded from the analyses.
Replication	The number of replicates for each specific experiment is indicated throughout the manuscript text, figure legends and methods. All attempts of replication were successful.
Randomization	Samples were separated into groups based on developmental age. Where possible, groups were evenly divided between male and female samples. All samples were processed in parallel.
Blinding	There was no blinding in this study prior to running the samples through the mass spectrometer because there were no treated or untreated groups, and intervention bias was not an issue given the nature of the study. Hierarchical clustering and statistical analyses performed following mass spectrometry are inherently unbiased approaches.<br>Immunofluorescent staining was performed in a blinded manner, as each pancreas was coded with a unique sample number and processed, sectioned, stained, imaged and quantified before identifying the donor age group.

Reporting for specific materials, systems and methods

We require information from authors about some types of materials, experimental systems and methods used in many studies. Here, indicate whether each material, system or method listed is relevant to your study. If you are not sure if a list item applies to your research, read the appropriate section before selecting a response.

Materials & experimental systems

n/a	Involved in the study
[ ]	[x] Antibodies
[x]	[ ] Eukaryotic cell lines
[x]	[ ] Palaeontology and archaeology
[x]	[ ] Animals and other organisms
[ ]	[x] Human research participants
[x]	[ ] Clinical data
[x]	[ ] Dual use research of concern

Methods

n/a	Involved in the study
[x]	[ ] ChIP-seq
[x]	[ ] Flow cytometry
[x]	[ ] MRI-based neuroimaging

Antibodies

Antibodies used

All antibodies used and product information are extensively detailed in Supplemental Table 8.

CD31 monoclonal; Raised in mouse; 1:50; Santa Cruz (sc-376764)
Collagen I polyclonal; Raised in rabbit; 1:500; Abcam (ab34710)
Collagen II polyclonal; Raised in rabbit; 1:200; Abcam (ab34712)
Collagen III polyclonal; Raised in rabbit; 1:500; Abcam (ab7778)
Collagen IV polyclonal; Raised in rabbit; 1:300; Abcam (ab6586)
Collagen V polyclonal; Raised in rabbit; 1:100; Abcam (ab7046)
Collagen VI polyclonal; Raised in rabbit; 1:200; Proteintech (17023-1-AP)
Collagen XII polyclonal; Raised in rabbit; 1:100; Invitrogen (PA5-38890)
Collagen XIV polyclonal; Raised in rabbit; 1:150; Invitrogen (PA5-54886)
Collagen XVI polyclonal; Raised in rabbit; 1:100; Sigma-Aldrich (SAB4500398)
EMILIN1 monoclonal; Raised in mouse; 1:100; Proteintech (60047-1-Ig)
FBN2 polyclonal; Raised in rabbit; 1:200; Proteintech (20252-1-AP)

FBN2 polyclonal; Raised in rabbit; 1:200; Proteintech (20252-1-AP)
FBN2 polyclonal; Raised in rabbit; 1:200; Proteintech (20252-1-AP)
Fibronectin polyclonal; Raised in rabbit; 1:200; Abcam (ab2413)
Mouse IgG1, Kappa Monoclonal [B11/6] - Isotype Control; 1:100; Abcam (ab91353)
Rabbit IgG, monoclonal [EPR25A] - Isotype Control; 1:100; Abcam (ab172730)
Laminin alpha 4 monoclonal; Raised in mouse; 1:200; Abcam (ab242198)
Laminin alpha 5 monoclonal; Raised in mouse; 1:100; Abcam (ab77175)
Mimecan polyclonal; Raised in rabbit; 1:50; Proteintech (12755-1-AP)
Periostin polyclonal; Raised in rabbit; 1:200; Proteintech (19899-1-AP)
Insulin monoclonal; Raised in mouse; Sigma-Aldrich (I2018)
Insulin polyclonal; Raised in guinea pig; Sigma-Aldrich (I8510)
Glucagon monoclonal; Raised in mouse; 1:1000; Sigma-Aldrich (G2654)
Somatostatin monoclonal; Raised in mouse; 1:50; Santa Cruz Biotechnology (sc-74556)

Validation

Antibody validation is detailed in Supplemental Table 8.

[#sc-376764] Specific for an epitope mapping between amino acids 699-727 at the C-terminus of PECAM-1 of mouse origin. Selected Validation Data available on supplier website and in previous publications.
[#ab34710] Specificity Statement: This antibody reacts with most mammalian Type I collagens and has negligible cross-reactivity with Type II, III, IV, V or VI collagens. Non-specific cross-reaction of anti-collagen antibodies with other human serum proteins or non-collagen extracellular matrix proteins is negligible.
[#ab34712] Specificity Statement: This antibody reacts with Type II collagens and shows negligible (less than 1%) cross reactivity with Type I, III, IV, V or VI collagens. Nonspecific cross reaction with other human serum proteins or non collagen extracellular matrix proteins is negligible.
[#ab7778] Specificity Statement: This type specific collagen antibody only recognizes 3D epitopes. Negligible cross-reactivity with Type I, II, IV, V or VI collagens. Non-specific cross reaction of anti-collagen antibodies with other human serum proteins or non-collagen extracellular matrix proteins is negligible
[#ab6586] Specificity Statement: Negligible cross-reactivity with Type I, II, III, V or VI collagens. Non-specific cross reaction of anticollagen antibodies with other human serum proteins or non-collagen extracellular matrix proteins is negligible.
[#ab7046] Specificity Statement: Negligible cross-reactivity with Type I, II, III, IV or VI collagens. Non-specific cross reaction of anticollagen antibodies with other human serum proteins or non-collagen extracellular matrix proteins is negligible.
[#17023-1-AP] Selected Validation Data available on supplier website
[#PA5-38890] Immunogen: A synthetic peptide derived from the internal region of human Collagen XII alpha1. Selected Validation Data available on supplier website
[#PA5-54886] Immunogen sequence: LLVTPTSGGKTNQLNLQNTATKAIIQGLMPDQNYTVQIIAYNKDKESKPAQGQFRIKDLEKRKDPKPR. Selected Validation Data available on supplier website
[#SAB4500398] The antiserum was produced against synthesized peptide derived from human Collagen XVI alpha1. Selected Validation Data available on supplier website
[#60047-1-Ig] Selected Validation Data available on supplier website
[#20252-1-AP] Selected Validation Data available on supplier website
[#G2654] Monoclonal Anti-Glucagon reacts with pancreatic glucagon in RIA and immunocytochemistry. The affinity constant of 6.1 x 10(8) L/M in RIA. The antibody weakly cross-reacts with gut glucagon (enteroglucagon) in an immunohistological assay. Cross-reactivity has been observed with glucagon-containing cells in fixed sections of pancreas from human, porcine, dog, rabbit, mouse, rat, guinea pig, and cat.
[#sc-74556] Selected Data and Citations available on supplier website
[#ab2413] Selected Validation Data available on supplier website
[#ab91353] Selected Validation Data available on supplier website
[#ab172730] Selected Validation Data available on supplier website
[#ab242198] Immunogen: Recombinant fragment corresponding to Human Laminin alpha 4/LAMA4 aa 670-809. Sequence: ENLLNQARELQAKAESSSDEAVADTSRRVGGALARKSALKTRLSDAVKQLQAAERGDAQQRLGQSRLITEEANRTTMEVQQATAPMANNLTNWSQNLQHFDSSAYNTAVNSARDAVRNLTEVVPQLLDQLRTVEQKRPAS. Selected Validation Data available on supplier website
[#ab77175] Immunogen: Full length native protein (purified) corresponding to Human Laminin alpha 5/LAMA5. Selected Validation Data available on supplier website
[#12755-1-AP] Selected Validation Data available on supplier website
[#19899-1-AP] Selected Validation Data available on supplier website
[#I2018] The antibody reacts specifically against insulin by RIA and immunocytochemistry. It exhibits cross-reactivity with human proinsulin. The antibody binds to insulin with an affinity constant of 8.8 × 109 M-1 in RIA.
[#I8510] Selected Validation Data available on supplier website and in previous publications.

Human research participants

Policy information about studies involving human research participants

Population characteristics	Postnatal pancreas: University of Wisconsin-Madison Health Sciences Institutional Review Board approved the use of postmortem human tissues for this study (waived from IRB protocol for post-natal tissues). This is not considered human subjects research. Donors were selected by age group and sex to create a bank of donors in each age category that were roughly half men and half women.<br>Fetal pancreas: The only donor characteristics we receive for these donors are gestational age and sometimes the sex if it can be determined, but often is not determined. The 18-20 gestational week age range was selected based on availability of tissues.
Recruitment	Postnatal pancreas: University of Wisconsin-Madison Health Sciences Institutional Review Board approved the use of postmortem human tissues for this study (waived from IRB protocol for post-natal tissues). This is not considered human subjects research. Donor organs were accepted for use in this study based on having no history of diabetes or pancreatitis, and no current signs of diabetes (normal Hba1c) and normal levels of serum lipase and amylase.<br>Fetal pancreas: Limited donor information is available for these tissues, donors were selected simply based on availability and gestational age.
Ethics oversight	Human pancreas tissue preparation. Human fetal pancreas tissue was obtained from secondary sources (Advanced Biosystems Resources, Inc.) under approved Material Transfer Agreements and with protocols approved by the University of Wisconsin's Institutional Animal Care and Use Committee (IACUC) and Institutional Review Board (IRB) (IRB Study #2013-141). ABR, Inc obtains consent in accordance with Uniform Anatomical Gift Act (UAGA) and National Organ Transplant Act (NOTA) guidelines. ABR, Inc warrants that appropriate consent for tissue donation is obtained and adequate records of such consents are maintained. In addition, that tissues are obtained with local, state, and federal laws and regulations governing the procurement of human tissue.<br><br>Juvenile and adult human pancreas tissue was procured by the University of Wisconsin Organ and Tissue Donation Services from donors with no indication of diabetes or pancreatitis, with consent obtained for research from next of kin and authorization by the University of Wisconsin-Madison Health Sciences Institutional Review Board (IRB granted an exempt from protocol approval for studies on post-natal tissue because research on deceased donors is not considered human subjects research). IRB oversight of the project is not required because it does not involve human subjects as recognized by 45 CFR 46.102(f) which defines a 'human subject' as "a living individual about whom an investigator (whether professional or student) conducting research obtains (1) data through intervention or interaction with the individual, or (2) identifiable private information."

Note that full information on the approval of the study protocol must also be provided in the manuscript.
